# *Pseudomonas putida*—a versatile host for the production of natural products

**DOI:** 10.1007/s00253-015-6745-4

**Published:** 2015-06-23

**Authors:** Anita Loeschcke, Stephan Thies

**Affiliations:** Institut für Molekulare Enzymtechnologie, Heinrich-Heine-Universität Düsseldorf, Forschungszentrum Jülich, 52426 Jülich, Germany; Cluster of Excellence on Plant Sciences (CEPLAS), Heinrich-Heine-Universität Düsseldorf, Universitätsstraße 1, 40225 Düsseldorf, Germany; Bioeconomy Science Center (BIOSC), c/o Forschungszentrum Jülich, 52425 Jülich, Germany

**Keywords:** *Pseudomonas putida*, Heterologous pathway expression, Recombinant biosynthesis, Natural products

## Abstract

The biosynthesis of natural products by heterologous expression of biosynthetic pathways in amenable production strains enables biotechnological access to a variety of valuable compounds by conversion of renewable resources. *Pseudomonas putida* has emerged as a microbial laboratory work horse, with elaborated techniques for cultivation and genetic manipulation available. Beyond that, this bacterium offers several particular advantages with regard to natural product biosynthesis, notably a versatile intrinsic metabolism with diverse enzymatic capacities as well as an outstanding tolerance to xenobiotics. Therefore, it has been applied for recombinant biosynthesis of several valuable natural products. This review provides an overview of applications of *P. putida* as a host organism for the recombinant biosynthesis of such natural products, including rhamnolipids, terpenoids, polyketides and non-ribosomal peptides, and other amino acid-derived compounds. The focus is on de novo natural product synthesis from intrinsic building blocks by means of heterologous gene expression and strain engineering. Finally, the future potential of the bacterium as a chassis organism for synthetic microbiology is pointed out.

## Introduction

For the biotechnological production of natural products, engineered bacteria generally offer several advantages over the original producers. As opposed to many natural producers, typically applied engineered bacteria are characterized by easy handling regarding laboratory cultivation which is the prerequisite for biotechnological applications. In addition, independence of natural regulation systems usually immanent in the original producer permits controlled biosynthesis and the construction of hyper-production strains. Furthermore, usage of non-harmful generally recognized as safe (GRAS)-certified strains such as *Pseudomonas putida* KT2440 allows studies in many laboratories as well as industrial-scale production. Applying well-established and genetically accessible laboratory work horses moreover allows for genetic manipulation of biosynthetic modules in order to direct biosynthesis to desired compounds.

*P. putida* has emerged as one of the laboratory work horses matching the abovementioned advantages and offering specific features of particular interest beyond that. In recent decades, this Gram-negative soil bacterium has been virtually “domesticated” by means of synthetic biology, as excellently reviewed by Nikel et al. (2014). Plenty of tools for genetic manipulation and gene expression are available including numerous inducible promoter systems (and corresponding inducer compounds) such as native Pm/XylS (*m*-toluate) (de Lorenzo et al. 1993), PsaI/NahR (salicylate) (de Lorenzo et al. 1993), and P*alkB*/AlkS (short-chain alkanes) (Panke et al. 1999) as well as the non-native systems P_*lac*_ (IPTG) (Baumberg et al. 1980), P_*tac*_ (IPTG) (Bagdasarian et al. 1983), P_*T7*_ (IPTG via P_*lac*_) (Troeschel et al. 2012), P_*T7*_ (*m*-toluate via Pm/XylS) (Herrero et al. 1993), NagR/P*nag*Aa (salicylate) (Hüsken et al. 2001), *rha*P_BAD_ (Rhamnose) (Jeske and Altenbuchner 2010), and P_*tet*_ (tetracycline) (Chai et al. 2012). The genomes of important strains such as *P. putida* KT2440 (Nelson et al. 2002) or S12 (Kuepper et al. 2015) are fully sequenced, providing the basis for understanding metabolic networks (Nelson et al. 2002; Puchałka et al. 2008; Wu et al. 2011) and sophisticated strain development approaches (Martínez-García et al. 2014b). Due to its relatively high guanine-cytosine (GC) content (61.5 %), *P. putida* is suitable for heterologous expression of genes from GC-rich bacterial clades like actinobacteria or myxobacteria that are especially rich in secondary metabolite biosynthesis gene clusters. On the level of biosynthesis, *P. putida* offers a wealth of cofactors especially for oxidoreductases (Blank et al. 2010; Tiso et al. 2014) and a versatile metabolism with diverse intrinsic enzymatic capacities for production purposes (Nelson et al. 2002), while at the same time, a rather “clean” background simplifies the detection of many heterologously synthesized metabolites (Martinez et al. 2004; Stephan et al. 2006). Moreover, the bacterium exhibits a high tolerance towards xenobiotics including antibiotics and organic solvents. This extraordinary feature is the result of complex adaptations such as effective efflux systems which are typically activated in presence of xenobiotics (Fernández et al. 2009; Simon et al. 2014), rendering it an ideal producer of such compounds and an especially suitable organism for production processes in two-phase system (Heipieper et al. 2007).

**Fig. 1:**
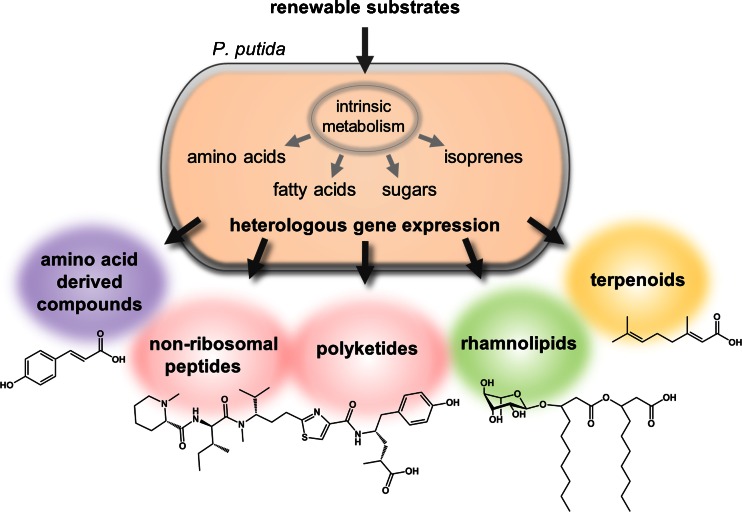
Hitherto described utilization of *Pseudomonas putida* as cell factory for the production of different natural products. Recombinant biosynthesis pathways are implemented using building blocks from intrinsic metabolism. Indicated examples are *from left to right p*-coumarate, pretubulysin A, mono-rhamnolipid, and geranic acid

This review covering the literature which has appeared until March 2015 aimed to summarize applications of *P. putida* as a host organism for recombinant natural product biosynthesis by means of heterologous gene expression and strain engineering. The focus is on bioconversion of basic nutrients for de novo synthesis of complex natural products from intrinsic building blocks (Fig. 1). Biotransformations, i.e., the turnover of preformed substrates into products by one or few recombinant biocatalysts (Berger 1995; Tiso et al. 2014), using *P. putida* as a host are important applications as well which have been recently excellently reviewed elsewhere (Poblete-Castro et al. 2012; Tiso et al. 2014) and are thus not covered by this review.

## Secondary metabolites of *P. putida*

Besides the utilization of *P. putida* for recombinant natural product biosynthesis, which is the main focus of this review, it is worth mentioning that strains of this organism naturally produce secondary metabolites of biotechnological interest. Particularly noteworthy are the polymers alginate (Chang et al. 2007; Tiso et al. 2014) and medium-chain length polyhydroxyalkanoates (PHA) (Valentin et al. 1998). The latter attracted special attention due to their physical and material properties; they are thermoplastic and/or elastomeric, are insoluble in water, are enantiomerically pure, are non-toxic, exhibit a high degree of polymerization, and are therefore discussed as alternative biodegradable polymers for biotechnological industry (Steinbüchel and Lütke-Eversloh 2003). This polyester of hydroxy fatty acids is synthesized by PHA synthase, an α/β-hydrolase-like enzyme, from R-3-hydroxyacyl-CoA which is supplied by several metabolic pathways (Rehm et al. 2001; Verlinden et al. 2007). The monomeric R-3-hydroxy fatty acids are in turn obtained from PHA hydrolysis to provide a chiral building block of pharmaceutical relevance (Lee et al. 1999; Yuan et al. 2008; O’Connor et al. 2013). Due to the material’s interesting properties, PHA synthesis was object of several studies concerning yield optimization and tailoring the polymer by metabolic engineering (Huijberts and Eggink 1996; Steinbüchel and Lütke-Eversloh 2003; Tiso et al. 2014; Vo et al. 2015).

Very recently, a rhizosphere soil bacterium identified as *P. putida* was shown to produce the antibiotic and antitumor phenazine derivate 5-methyl-phenazine-1-carboxylic acid (Kennedy et al. 2015).

Furthermore, different *P. putida* strains are known to produce non-ribosomal peptides (Gross and Loper 2009), which were, however, not yet intensely studied in terms of biotechnological applications. Very common among pseudomonads is the synthesis of the fluorescent siderophore pyoverdine, which is discussed for different applications such as plant growth promotion (Glick 2012; Saha et al. 2015). *P. putida* strains 267, PCL1445, and W15Oct28 or strain RW10S2 are moreover reported to release lipopeptide biosurfactants with antimicrobial properties, namely putisolvins and a viscosin-like peptide (Kuiper et al. 2004; Kruijt et al. 2009; Rokni-Zadeh et al. 2012; Ye et al. 2014). In silico genome mining of additional secondary metabolite clusters for *P. putida* KT2440 with antiSMASH 3.0 (Weber et al. 2015) revealed a gene cluster comprising another non-ribosomal peptide synthetase gene (cluster location 4,798,235–4,851,188 nt, NRP gene PP4243) and two clusters for biosynthesis of bacteriocins (1,126,477–1,137,310 and 2,738,633–2,749,466 nt), members of PiPPs (ribosomally synthesized and post-translationally modified peptides). An analogous analysis of the *P. putida* S12 chromosome and megaplasmid pTTS12 likewise unveiled the presence of two clusters for bacteriocin biosynthesis (951,794–962,627 and 4,313,737–4,324,570 nt), furthermore a type I polyketide synthase (3,653,938–3,701,437 nt, polyketide synthase (PKS) gene RPPX16405), but no further non-ribosomal peptide synthesis-related genes besides the cluster for pyoverdine synthesis.

**Fig. 2:**
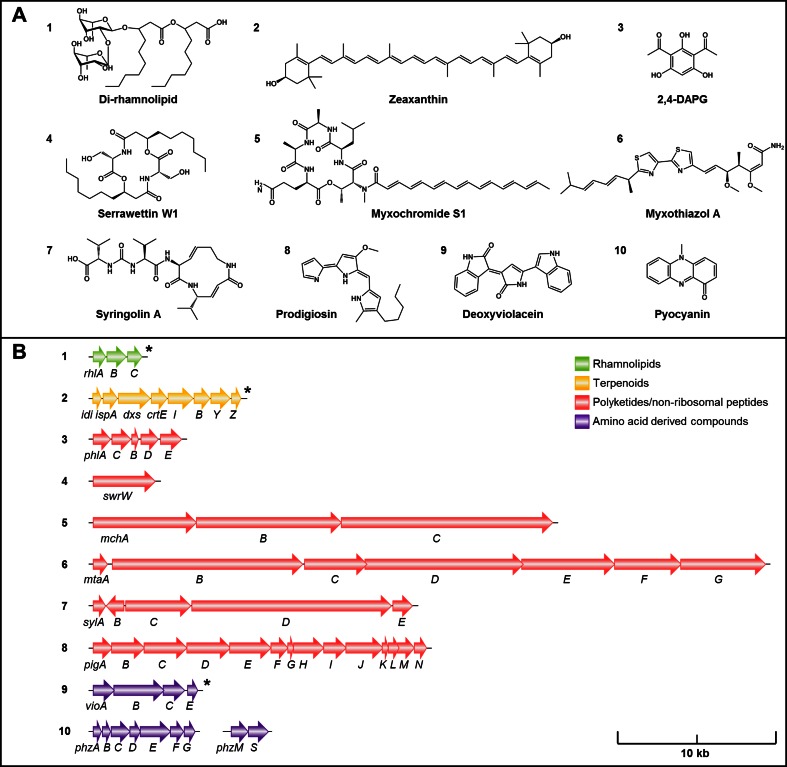
Representative examples of natural products synthesized in *P. putida.* **a** Product structural formula; **b** biosynthetic genes needed to produce compounds shown in **a**; numbering of gene clusters refers to compounds shown in **a,** *asterisk* indicates synthetic operons

In the following, different groups of natural products synthesized in recombinant *P. putida* will be discussed, namely rhamnolipids, terpenoids, polyketides and non-ribosomal peptides as well as hybrids thereof, and further amino acid-derived compounds (Fig. 1). All products together with relevant characteristics of the respective studies are summarized in Table 1; selected examples are shown in Fig. 2 to illustrate diversity of compounds and architecture of cognate biosynthetic gene clusters.

**Table 1:** Natural products synthesized in *P. putida* by heterologous gene expression and strain engineering

Product^a^	Native producer^b^		*P. putida* ^*c*^	Expression strategy^d^	Yield^e^	Reference
Rhamnolipids (RL)						
Mono-RL		*P. aeruginosa*	KT2442	P_*tac*_, *rhlAB* (2.2 kb), pl	0.60 g/l C yield 0.17	Ochsner et al. 1995
			KT2440	P_*tac*_, *rhlAB* (2.2 kb), pl	0.57 g/l	Setoodeh et al. 2014
			KT2440	P_*tac*_, *rhlAB* (2.3 kb), pl., *Δ*phaC1*	1.5 g/l C yield 0.23	Wittgens et al. 2011
			KT2440	P_tac_*/P_synthetic_^1^, *rhlAB* (2.3 kb), pl., * Δ*phaC1*	up to ^1^2.5 g/l *C yield 0.23	Blank et al. 2013
			KCTC 1067	P_native_(RhlRI), *rhlABRI* (4 kb), pl.	7.3 g/l C yield 0.17	Cha et al. 2008
			KT2440	P_native_(RhlRI), *rhlABRI* (4.5 kb), chr	1.68 g/l	Cao et al. 2012
Mono- and di-RL		*P. aeruginosa*	KT2440/GPp104	*rhlAB*/*rhlABM* (2.3 kb/3.6 kb syn op), *rhlABC*/*rhlABMC* (3.3 kb/4 kb syn op), pl	di-RL: 113 mg/l/OD_600_	Schaffer et al. 2012
Mono- and di-RL		*B. glumae*	KT2440	P_*tac*_, *rhlAB*(*C*) (3.4 kb), pl	80 mg/l (mono-RL), 50 mg/l (mixture)	Blank et al. 2013
Terpenoids						
Geranic acid		*O. basilicum*	DSM 12264	*rha*P_BAD_, *ges* + *MVA genes of *M. xanthus* (~8.5 kb syn op), pl	193 mg/l, BR FB	Mi et al. 2014
Zeaxanthin		*P. ananatis*	KT2440	*rha*P_BAD_, *crtEIBYZ* + *isoprenoid genes of *E. coli* (~8.5 kb syn op), pl	239 mg/l, FB	Beuttler et al. 2011
β-Carotene Zeaxanthin		*P. ananatis*	KT2440	P_*T7*_, *crtE*Δ*XYIBZ*, (6.9 kb), chr	0.2 mg/gCDW	Loeschcke et al. 2013
Polyketides/Non-ribosomal peptides						
2,4-DAPG		*P. fluorescens*	KT2440	P_native_/P_chr of *P. putida*_, *phlACBDE* (6.5 kb), chr	n.d.	Martinez et al. 2004
Flaviolin		*S. cellulosum*	KT2440	Pm, *rppA* (1.1 kb), pl.	~6 mg/l	Gross et al. 2006a
β-Lactam DAC		*L. lactamgenus*	IFO14164	P_*lac*_, *pcbABCcefEFDbla* (16 kb), pl	~2 mg/l	Kimura et al. 1996
Serrawettin W1		*S. marcescens*	KT2440	P_*tac*_, *swrW* (4 kb), pl	n.d.	Thies et al. 2014; unpublished
Myxochromide S		*S. aurantiaca*	KT2440	Pm, *mchABC* (30 kb), chr	40 mg/l	Wenzel et al. 2005
Myxothiazol A		*S. aurantiaca*	KT2440	Pm, *mtaBCDEFG* (60 kb), chr, *mm-CoA^+^	0.6 mg/l	Gross et al. 2006b, Perlova et al. 2006
(Tyrosin) Pretubulysin A		*Cystobacter* sp.	KT2440	P_native_/P_*tet*_, *tubAorf2tubZorf1tubBCDEForf17orf18* (~40 kb), chr	1.76 μg/l	Chai et al. 2012
Syringolin A		*P. syringae*	P3	P_native_, *sylABCDE* (22 kb), cos	n.d.	Ramel et al. 2009
Glidobactin A		*P. luminescens*	P3	P_native_, *plu1881-1877* (19 kb), cos	n.d.	Dudnik et al. 2013
Prodigiosin		*S. marcescens*	KT2440	P_*T7*_, *pigABCDEFGHIJKLMN* (22 kb), chr	0.5 mg/gCDW	Loeschcke et al. 2013
Amino acid-derived compounds						
Phenol		*P. agglomerans*	S12	NagR/pNagAa, *tpl* (1.4 kb), pl., *random mutagenesis	9.2 mM, C yield 0.07 biphasic BR FB	Wierckx et al. 2005
*t*-Cinnamate		*R. toruloides*	S12	P_*tac*_, *pal* (2.5 kb), pl., *random mutagenesis	5.4 mM, BR FB C yield 0.07	Nijkamp et al. 2005
*p*-Coumarate		*R. toruloides*	S12	P_*tac*_, *pal* (2.5 kb), pl., *random muta-genesis: Phe auxotrophy, Δ*fcs*	1.7 g/l, BR FB C yield 0.04	Nijkamp et al. 2007
*p*-Hydroxy-styrene		*R. toruloides* *L. plantarum*	S12	NagR/pNagAa, *pal pdc* (3 kb syn op), pl., *Δ*fcs* Δ*smo*	21 mM, Cmol 0.04, biphasic BR FB	Verhoef et al. 2009
*p*-Hydroxy-benzoate		*R. toruloides*	S12	P_*tac*_, *pal* (2.5 kb), pl., *Δ*pobA*	1.8 g/l, BR FB C yield 0.11	Verhoef et al. 2007
*p*-Hydroxy-benzoate		*R. toruloides*	S12	P_*tac*_, *pal* (2.5 kb), pl., *Δ*pobA*, Δ*hpd*	2.3 mM C yield 0.13	Verhoef et al. 2010
*p*-Hydroxy-benzoate		*R. toruloides*	S12	P_*tac*_, *pal* (2.5 kb), pl., *Δ*gcd*, *xylAB*_*FGH* ^+^	C-yield 0.16 chemostat	Meijnen et al. 2011b
Deoxyviolacein		*Duganella* sp.	mt−2	P_*alkB*_, *vioABCE* (6.2 kb syn op), pl	1.5 g/l	Xing and Jiang 2011
*N*-Acyl aromatic amino acid		*Metagenome*	KT2440	eDNA fragment of 28.8 kb, cos	n.d.	Craig et al. 2010
Phenazine PCA		*P. fluorescens*	WCS358r	P_*tac*_, *phzABCDEFG* (6.8 kb), chr	n.d.	Glandorf et al. 2001
Pyocyanin		*P. aeruginosa*	KT2440	NagR/pNagA, *phzA1B1C1D1E1F1G1* (6.7 kb), *phzMS* (2.2 kb syn op), pl.	45 mg/l	Schmitz et al. 2015
MEA		*A. thaliana*	S12	NagR/pNagA, *sdc*-tr (1.3 kb), pl., *Δ*eutBC*	2.6 mM	Foti et al. 2013
Cyanophycin		*Synechocystis* sp.	KT2440^2^/ GPp104	P_native_/P_*lac*_, *cphA*, (3.3 kb), pl	^2^11 % of CDW	Aboulmagd et al. 2001
		*Synechocystis* sp.	KT2440/GPp104^3^	P_native_/P_*lac*_, *cphA* (3.3 kb), pl.	^3^10 % of CDW	Voss et al. 2004
		*Anabaena* spec.	KT2440/GPp104^4^	P_native_/P_*lac*_, *cphA1* (3.3 kb), pl	^4^24% of CDW	Voss et al. 2004
		*Synechocystis* sp.	KT2440/GPp104^5^	P_native_/P_*lac*_, *cphA* (3.2 kb), pl	^5^9.7 % of CDW	Voss et al. 2004
		*Synechococcu*s spec.	KT2440/GPp104^6^	P_native_/P_*lac*_, *cphA* (3 kb), pl	^6^17.5 % of CDW	Voss et al. 2004
Citrulline-cyanophycin		*Synechocystis* sp.	ATCC 4359	P_native_/P_*lac*_, *cphA*, (3.3 kb), pl	43.4 % of CDW	Wiefel et al. 2011

Synthesized products (^a^) are listed together with the native producers whose corresponding biosynthetic genes were employed (^b^). The *P. putida* strains used for production (^c^) and the respective expression strategies (^d^) are indicated. Here, applied promoters, expressed genes and their size, as well as the mode of maintenance within the host are named: plasmid (pl.), cosmid (cos), or chromosome (chr). Additional strain engineering is indexed (*). Product yields are given in units as stated in the original publications, carbon yield (Cmol_product_/Cmol_substrate_) is abbreviated as C yield, if stated (^e^). The applied production processes other than shake flask batch cultivations are indicated.

*FB* fed batch, *BR* bioreactor cultivation

### Rhamnolipids

Rhamnolipids are currently considered as the best studied representatives of bacterial biosurfactants (Müller et al. 2012), a class of metabolites with most various chemical compositions produced by different bacteria and fungi (Hausmann and Syldatk 2014). Due to excellent surfactant properties, low toxicity, high biodegradability, and antimicrobial effects, rhamnolipids are discussed for various applications, e.g., in cleaning agents, cosmetics, food industry, biocontrol, and soil remediation (Fracchia et al. 2014). Rhamnolipids consist of a hydrophobic domain with generally two molecules of hydroxy fatty acids forming 3-(hydroxyalkanoyloxy)alkanoic acid (HAA) and a hydrophilic part of one or two molecules of the sugar rhamnose, thus forming mono-rhamnolipids and di-rhamnolipids, respectively (Abdel-Mawgoud et al. 2010). At least two enzymes are required for the biosynthesis of rhamnolipids from the precursor metabolites dTDP-rhamnose and hydroxy fatty acid-ACP: (i) the acyltransferase RhlA for generation of HAA and (ii) the rhamnosyltransferase RhlB for glycosidic bond formation. For synthesis of di-rhamnolipids, a second molecule of activated rhamnose is added to mono-rhamnolipids by the rhamnosyltransferase RhlC (Abdel-Mawgoud et al. 2011).

The opportunistic human pathogen *Pseudomonas aeruginosa* is the best-studied organism for rhamnolipid production*.* Although nowadays different non-pathogenic bacteria are described to be capable of rhamnolipid formation (Toribio et al. 2010), among them the β-proteobacterium *Burkholderia glumae* (Costa et al. 2011; Voget et al. 2015), the marine *Thermus* sp. (Rezanka et al. 2011), and some strains from the *P. putida* group (Tuleva et al. 2002; Martínez-Toledo et al. 2006), most studies concerning optimization of rhamnolipid production are performed with *P. aeruginosa* (Müller et al. 2010; Müller et al. 2012). Recombinant expression of rhamnolipid biosynthesis pathways in closely related *P. putida* offers a promising alternative.

**Mono-rhamnolipid** (Fig. 1) production in *P. putida* strains by expression of *rhlAB* from diverse strains of *P. aeruginosa* and the product’s extracellular accumulation was reported by several groups at gram scale. Different promoters were evaluated for expression of the ca. 2-kb operon, namely the synthetic hybrid promoter P_*tac*_, (Ochsner et al. 1995; Wittgens et al. 2011; Setoodeh et al. 2014), a set of completely synthetic promoters in comparison to P_*tac*_ (Blank et al. 2013), or the native regulation system via coexpression of the cognate autoinducer-dependent transcription factor/autoinducer synthase pair RhlR/RhlI from *P. aeruginosa* (Cha et al. 2008; Cao et al. 2012). Metabolic network analysis identified the PHA formation (see above) via PHA synthases PhaC1/C2 as a competitor for the precursor hydroxy fatty acid-ACP. Consequential deletion of *phaC1* was proven to be beneficial for rhamnolipid product accumulation (Wittgens et al. 2011).

Remarkably, a rhamnolipid-producing *P. putida* strain with genome-integrated *rhlABRI* could be applied for remediation of soil contaminated with PAH (polycyclic aromatic hydrocarbons), whereat interestingly *P. putida* itself was shown to be unable to degrade PAH, but the secreted biosurfactants vastly increased the degradation by native soil microorganisms (Cao et al. 2012).

Mono-rhamnolipids can be easily converted to **di-rhamnolipids** (Fig. 2, **1**), as described above, via the activity of the second rhamnosyltransferase RhlC to form more hydrophilic biosurfactants. Nonetheless, there are only two reports describing the heterologous expression of all three RL-synthesis genes in *P. putida*. Expression of *rhlABC* from *P. aeruginosa* yielded up to 113 mg/l/OD_600_ using PHA-deficient *P. putida* GPp104 (Schaffer et al. 2012). Notably, di-rhamnolipid concentration in the culture supernatant was increased when PA1131, a putative major facilitator superfamily transporter that is organized in one operon with *rhlC*, was coexpressed. Thus far, the mechanism of rhamnolipid secretion is described neither for heterologous production strains nor for the native producers, and it remains unclear if PA1131 contributes actively to the secretion of the products or if there are currently unknown regulatory effects. Blank et al. (2013) demonstrated that rhamnolipid properties may vary with the origin of the biosynthetic genes applied. They reported the production of both mono- and di-rhamnolipids expressing *rhlAB*(*C*) genes from *B. glumae* PG1 controlled by P_*tac*_, yielding 80 mg/l of pure mono-rhamnolipids and 50 mg/l of a mixture, respectively. Compared to *P. aeruginosa*, *B. glumae* naturally produces more hydrophobic rhamnolipids with higher average fatty acid chain length. Remarkably, comparable fractions were produced upon expression of the respective genes in *P. putida* KT2440.

The here reviewed studies from both academia and industry illustrate that *P. putida* has emerged as an excellent platform for recombinant rhamnolipid production and may thus replace the pathogenic *P. aeruginosa* in future rhamnolipid production processes. An additional advantage of *P. putida* is its resistance to very high concentrations of rhamnolipids showing little change in growth rate if exposed to concentrations up to 90 g/l (Wittgens et al. 2011), suggesting that even higher yields are feasible. Crucial factors for optimization of recombinant rhamnolipid production in *P. putida* include the choice of an appropriate promoter and the increase in biosynthetic flux toward the product, e.g., by deletion of the competing PHA pathway.

### Terpenoids

Terpenoids constitute one of the most diverse groups of secondary metabolites in nature, and many of them find applications in areas such as human health and nutrition. They are synthesized from universal C_5_ isoprene precursors isopentenyl diphosphate (IPP) and its isomer dimethylallyl diphosphate (DMAPP). Terpene synthases cyclize poly-isoprene units or produce linear compounds thereof. Typically, the synthase reaction is followed by decoration of the molecule, carried out by terpene-modifying enzymes such as cytochrome P450 monooxygenases (Bouvier et al. 2005; Cane and Ikeda 2012). Much effort has been devoted toward their production in microbial hosts (Kirby and Keasling 2009; Li and Pfeifer 2014), mostly employing *Escherichia coli* and yeast.

The production of terpenes using *P. putida* started with biotransformation approaches, aiming to yield oxidation products of the plant monoterpene limonene which was supplemented to the process. These products are valuable for their flavoring, antibiotic, and anticancer properties (Schrader 2007; Garcia et al. 2010). In one setup, a heterologously expressed P450 was applied for the hydroxylation of (*S*)-limonene to (*S*)-perillyl alcohol (Van Beilen et al. 2005; Cornelissen et al. 2013). Other studies exploited the bacterium’s intrinsic enzymes that naturally degrade *p*-cymene via *p*-cumate. This three-step degradation involves enzymes also active towards limonene and its respective oxidation products perillyl alcohol and perillaldehyde, thereby converting limonene to the monoterpenoid perillic acid (Speelmans et al. 1998; Mars et al. 2001; Mirata et al. 2009).

Full de novo biosynthesis of a monoterpenoid was demonstrated recently (Mi et al. 2014). In this study, *P. putida* strain DSM12264 was used for the production of *geranic acid* (Fig. 1) which has received attention for potential applications in fragrance and flavor industries (Schrader 2007). It was achieved by expression of geraniol synthase from *Ocimum basilicum* (basil) whose product geraniol was converted to geranic acid by an intrinsic enzyme. The required C_10_ substrate for the synthase is geranyl pyrophosphate (GPP). It is provided by *P. putida* via the MEP (2-*C*-methylerythritol-4-phosphate) isoprenoid biosynthesis pathway which starts using pyruvate and glyceraldehyde-3-phosphate from central metabolism and is the common pathway in most bacteria. Yields were improved significantly by increasing the isoprene precursor pool by coexpression of the six genes of the MVA (mevalonate) isoprenoid biosynthesis pathway from the myxobacterium *Myxococcus xanthus* DSM16526 (*hmgs*, *hmgr*, *mvk*, *pmvk*, *mvd*, and *idi*). This way, a second route from central metabolism (acetyl-CoA) to isoprenoid building blocks was installed. All genes were assembled into a synthetic operon (of about 8.5 kb) under control of the *rha*P_BAD_ promoter from *E. coli* in one plasmid. In a lab-scale bioreactor setup, 193 mg/l of geranic acid could be produced. Notably, this is the first report showing feasibility of microbial production of this compound in general. The product was for the most part found in the supernatant, whereby downstream processing is facilitated. Moreover, in associated toxicity tests, the authors could show that *P. putida* exhibited tolerance to significantly higher concentrations of the product than *E. coli* and yeast. This underlines the potential of *P. putida* as production host for monoterpenoids.

Besides these examples for biosynthesis of monoterpenoids, recombinant production of tetraterpenes was also established using *P. putida*. The production of the yellow-colored xanthophyll carotenoid *zeaxanthin* (Fig. 2, **2**), which is relevant as food and feed additive and offers potential in pharmaceutical applications (Baiao et al. 1999; Nishino et al. 2009; Abdel-Aal et al. 2013), was reported by Beuttler et al. (2011). Expression of five carotenoid biosynthesis genes (*crtEBIYZ*) from the enterobacterium *Pantoea ananatis* DSM30080 established the conversion of the C_15_ isoprenoid precursor farnesyl pyrophosphate (FPP) to zeaxanthin. In addition, three genes from the *E. coli* MEP pathway (*idi*, *ispA*, and *dxs*) were coexpressed in order to enhance metabolic flux from C_5_ isoprenoid building blocks via GPP to FPP. All genes were assembled in an expression vector as a synthetic operon of about 8.5 kb under control of the *rha*P_BAD_ promoter and expressed in *P. putida* KT2440. After optimization of cultivation conditions and media additives, 239 mg/l of zeaxanthin could be produced. The same *crt* genes were used in another study for heterologous zeaxanthin biosynthesis in *P. putida* KT2440 (Loeschcke et al. 2013). Here, the applied transfer and expression system named TREX enabled the direct expression of the bidirectional natural gene cluster by convergent transcription from two T7 promoters, thereby circumventing the need for genetic reengineering. Chromosomal integration of different deletion versions of the cluster and subsequent T7 RNA polymerase-dependent expression led to production of zeaxanthin and *β-carotene*. Notably, this setup without engineered enhancement of the precursor pool only generated yields at low milligram scale.

The suitability of *P. putida* as terpenoid producer is hard to evaluate based on the low number of available studies. However, substantial yields of geranic acid and zeaxanthin suggest a promising potential. Engineering of the supply of isoprene precursors via the MEP or MVA pathway appears to be the crucial factor for success here.

## Polyketides/Non-ribosomal peptides

Polyketides and non-ribosomal peptides include a large and extremely diverse group of natural compounds with various highly valuable bioactivities such as antibiosis and cytotoxicity. They share features in biosynthesis and often cooccur in hybrid assembly systems (Wang et al. 2014). Briefly, the biosynthetic proteins of both assembly machineries catalyze the condensation of simple building blocks, i.e., carboxylic or amino acids, to produce polymer chains that can be cyclized and decorated to form numerous natural products. Three different types of polyketide synthases (PKSs) produce carbonyl polymers by condensing activated acyls (typically acetyl-CoA and malonyl-CoA) (Shen 2003; Cummings et al. 2014): Type I PKSs are large, highly modular proteins containing domains which catalyze biosynthesis steps, whereas type II PKSs are complexes of multiple individual proteins with dedicated functions. In both types, the elongating polymer is handed from one polymer extending module to the next, where it is linked to the protein by thioester bonds. Type I and II systems share features and nomenclature with fatty acid synthases. Type III PKSs, also referred to as chalcone synthase-like PKSs, are homodimeric enzymes that catalyze condensation and cyclization reactions producing phenolic products. Non-ribosomal peptide synthases (NRPSs) produce peptidyl polymers by adenylation and subsequent condensation of amino acids. Like in polyketide synthesis, the elongating polymer is bound to the enzyme by a thioester bond. Similar to PKSs, there are both modular multidomain NRPS enzymes and NRPS enzyme complexes (Finking and Marahiel 2004; Strieker et al. 2010).

Heterologous production of polyketides and non-ribosomal peptides has been demonstrated employing a diverse set of hosts, including fungi, Gram-positive bacteria, and Gram-negative bacteria such as *M. xanthus*, *E. coli*, and *P. putida* (Fujii 2009; Zhang et al. 2011; Ongley et al. 2013). For heterologous expression of the described systems, it is important to consider that with exception of type III PKSs, PKSs and NRPSs require their acyl carrier protein (ACP) and peptidyl carrier protein (PCP) domains, respectively, to be posttranslationally modified by a phosphopantetheinyl transferase (PPTase) in order to function. The genes for cognate PPTases are often not part of the PKS/NRPS gene cluster. Notably, the predominantly used *P. putida* strain KT2440 provides a broad substrate range PPTase which is able to activate both ACP and PCP domains (Gross et al. 2005; Owen et al. 2011) and is often more suitable than, e.g., *E.coli* PPTase, which circumvents the constraints of additional introduction of foreign PPTase genes.

### Polyketides

**Fig. 3:**
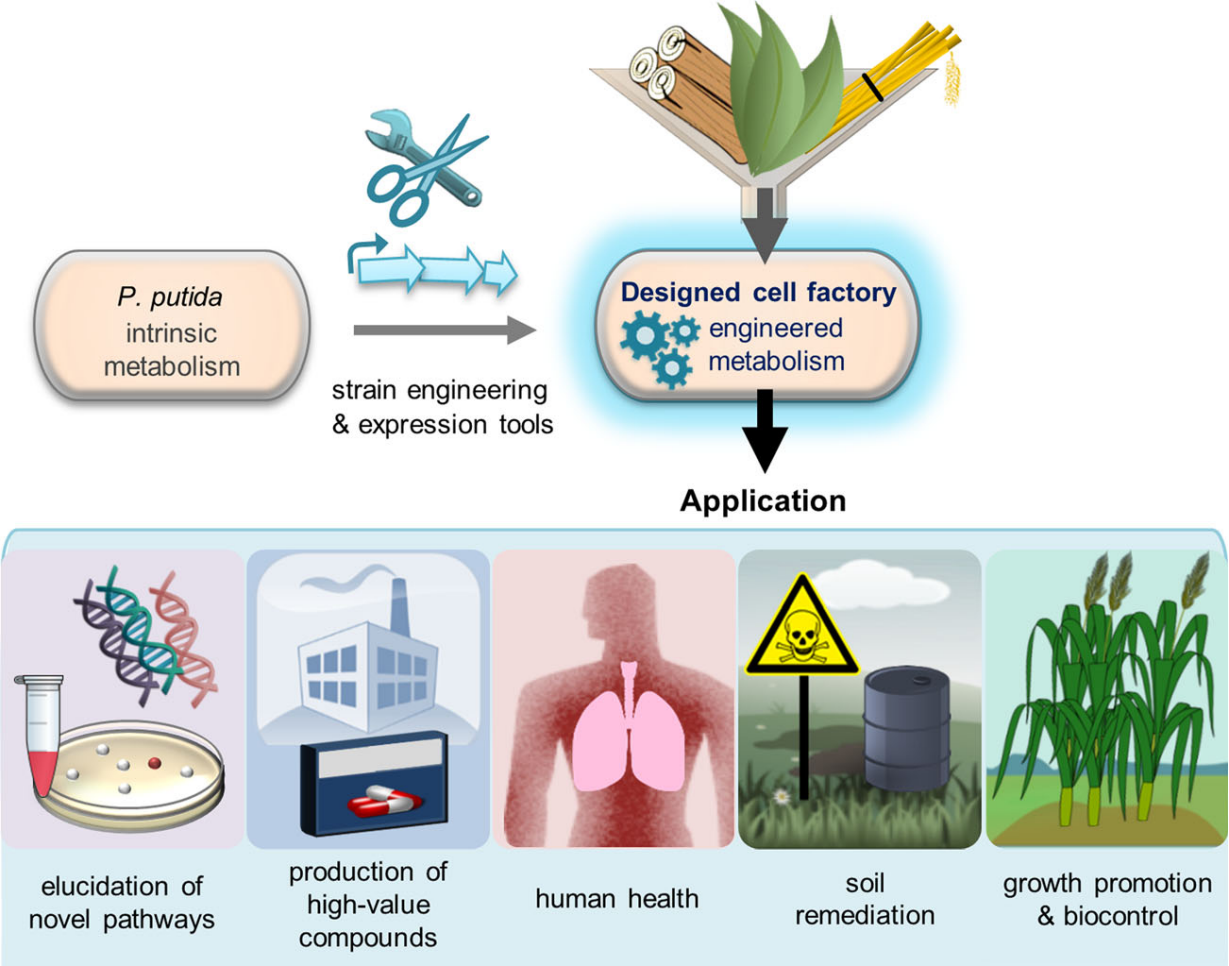
Perspectives in *P. putida* research and application. Newly developed sophisticated strain engineering and expression tools will generate next-generation designed *P. putida* cell factories able to convert various renewable substrates into a wealth of desired compounds with high precision and efficiency. This may enable highly diverse applications in the future: *P. putida* may be utilized for the identification and elucidation of natural product pathways as well as for the biotechnological production of high-value compounds. At the interface of synthetic microbiology and medicine, pharmaceutical application, e.g., of its outer membrane vesicles is suggested. Further, the bacterium may be applied in an ecological and agricultural context for remediation of soil, plant growth promotion, and biocontrol

The first polyketide heterologously produced in *P. putida* was 2,4-DAPG (*2,4*-*diacetylphloroglucinol*) (Fig. 2, **3**) which has received interest for its activity against plant pathogens (Bakker et al. 2002; Haas and Défago 2005) and methicillin-resistant *Staphylococcus aureus* (Kamei and Isnansetyo 2003). A 6.5-kb DNA fragment from *Pseudomonas fluorescens* ATCC 49323 containing the respective gene cluster was inserted into the genome of *P. putida* KT2440 (Martinez et al. 2004). It encompasses five unidirectionally organized genes (*phlACBDE*), where PhlD is a type III polyketide synthase catalyzing the synthesis of monoacetylphloroglucinol from three molecules of malonyl-CoA, which is in turn converted to 2,4-DAPG by the action of the other *phl*-encoded enzymes (Bangera and Thomashow 1999; Achkar et al. 2005). The expression of the genome-integrated *phl* cluster in *P. putida* was presumably driven by its native promoters or by chromosomal promoters adjacent to the insertion site. The accumulation of 2,4-DAPG could be detected but was not quantified.

Another type III PKS product, the UV protective pigment (Zeng et al. 2012) *flaviolin* (2,5,7-trihydroxy-1,4-naphthoquinone), could be synthesized in *P. putida* KT2440 (Gross et al. 2006a). Here, the 1.1-kb *rppA* gene from myxobacterium *Sorangium cellulosum* So ce56 was expressed from a plasmid using the *m*-toluate-inducible Pm promoter from *P. putida*. RppA, a 1,3,6,8-tetrahydroxynaphthalene synthase (THNS), utilizes five malonyl-CoA to synthesize THN, which is converted by autooxidation to flaviolin. About 6 mg of the red colored product could be purified from 1 l cell-free supernatant of culture broth.

### Non-ribosomal peptides

The earliest study showing the heterologous biosynthesis of a non-ribosomal peptide in *P. putida* reports on the production of the β-lactam antibiotic *deacetyl***-***cephalosporin C* (DAC). *P. putida* IFO14164 was used for expression of the clustered genes *pcbABCcefEFD* from the actinomycete *Lysobacter lactamgenus* YK90 (Kimura et al. 1996). The 16-kb gene region comprising five unidirectionally oriented genes was expressed from a plasmid under control of P_*lac*_ from *E. coli*. The *pcbAB* gene encodes one protein comprising three NRPS modules, which enabled the synthesis of LLD-ACV (δ-(l-α-aminoadipyl)-l-cysteinyl-d-valine). The non-canonic amino acid l-α-aminoadipic acid is derived from lysine and provided by *P. putida*. By the action of the other cluster-encoded enzymes, LLD-ACV is converted to the antibiotic DAC via penicillin N. DAC is naturally further differentiated to various β-lactam products (Demirev et al. 2006; Hamed et al. 2013). The *L. lactamgenus* specific end product cephabacin was not produced, since the hitherto required enzymes were excluded in the study. The study did not focus on production yield; however, about 2 mg of DAC could be extracted from cell material obtained from 1-l broth.

Recently, the biosynthesis of *serrawettin W1* (Fig. 2, **4**), another NRPS-derived compound, could be established in *P. putida*. This cyclic lipopeptide has gathered attention for its biosurfactant properties as well as antimicrobial and antitumor activities (Matsuyama et al. 2011; Kadouri and Shanks 2013). In order to produce the compound, the 4-kb *swrW* gene from the enterobacterium *Serratia marcescens* DSM12481 was cloned in a vector under control of P_*tac*_ (Thies et al. 2014). The SwrW protein comprises the NRPS module required to produce the symmetrical serrawettin W1 molecule consisting of two serine residues attached to two β-hydroxy fatty acids via ester and amide bonds. Expression of *swrW* in *P. putida* KT2440 enabled serrawettin W1 recovery from culture supernatant at milligram scale (S Thies, unpublished).

#### Polyketide/Non-ribosomal peptide hybrid compounds

The first product synthesized by a heterologously expressed type I PKS/NRPS hybrid system in *P. putida* was *myxochromide S* (Fig. 2, **5**) (Wenzel et al. 2005). To this end, the 30-kb *mchABC* cluster from *Stigmatella aurantiaca* DW4/3-1 was introduced into *P. putida* KT2440. The PKS module which utilizes acetyl-CoA, malonyl-CoA, and propionyl-CoA is encoded in *mchA*, while *mchB* encodes a two-module NRPS and *mchC* a four-module NRPS (one of those being skipped in biosynthesis), together forming the peptide part from alanine, glutamine, threonine, and leucine. The unidirectional gene cluster was inserted into the host chromosome at the anthranilate synthase gene *trpE* by homologous recombination. Expression of *mch* genes and thus accumulation of the yellow-orange colored myxochromide S in *P. putida* cells was implemented using the *m*-toluate-inducible Pm promoter. Notably, lowering of expression temperature from 30 to 16 °C after induction resulted in a 1000-fold increase of production to 40 mg/l. Supply of malonyl-CoA was identified as yield-limiting bottleneck that might be addressed in future studies to increase myxochromide production (Stephan et al. 2006).

Another myxobacterial type I PKS/NRPS hybrid system from *S. aurantiaca* DW4-3/1 expressed in *P. putida* resulted in the production of *myxothiazol A* (Fig. 2, **6**) (Perlova et al. 2006; Gross et al. 2006b), an inhibitor of the respiratory chain with antifungal and insecticidal activities (Clough 1993). The 60-kb *mta* gene cluster (*mtaBCDEFG*) was inserted into the chromosome of *P. putida* by the same method as the abovementioned *mch* cluster in gene *trpE*. While MtaB, MtaE, and MtaF are PKS parts and MtaC and MtaG are NRPS modules, the gene *mtaD* encodes a hybrid protein containing both PKS and NRPS modules. The PKS parts employ isovaleryl-CoA, which is provided by *P. putida* from the degradation of leucine, as the starter unit, and acetyl-CoA as well as methylmalonyl-CoA (mm-CoA) as extender units. The NRPS modules incorporate the amino acids cysteine and glycine (Perlova et al. 2006). Since *P. putida* does not intrinsically synthesize mm-CoA, the bacterium was engineered to provide the necessary precursor from its succinyl-CoA pool. This was achieved by expression of an operon from *S. cellulosum* So ce56 encoding mm-CoA epimerase (*epi*), mm-CoA mutase (*mcm*), and an MCM complex protecting protein (*meaB*) that was integrated in the chromosome. Expression of the mm-CoA operon was executed either by coexpression with the host genes at the integration site and/or driven by the promoter of a neomycin resistance gene which was coinserted. Expression of the myxothiazol cluster using the Pm promoter resulted in minor production levels. Product formation was increased to 0.6 mg/l by feeding of leucine and vitamin B12. Both the vitamin, required as cofactor for mm-CoA mutase, and the precursor leucine are intrinsically synthesized by *P. putida* but nevertheless appear to be limiting factors in product formation.

Via expression of the tubulysin gene cluster from the myxobacterium *Cystobacter* sp. SBCb004, a further type I PKS/NRPS hybrid system, was successfully installed in *P. putida* (Chai et al. 2012). The applied ~40 kb gene cluster comprised 11 genes, namely the unidirectional core assembly line part *tubBCDEF* as well as *tubA*, *tubZ*, *orf2*, *orf1*, *orf17*, and *orf18* organized around it (Sandmann et al. 2004). While TubB, TubC, and TubE are NRPS parts and TubF is a PKS part, TubD is a PKS/NRPS hybrid protein. First, TubZ produces pipecolic acid from lysine, which is then methylated and used as starter unit *N*-methyl-pipecolic acid by TubB. By extension with isoleucine, valine, cysteine, and tyrosine or phenylalanine as well as two molecules of acetyl-CoA, the core pretubulysin is formed, which is naturally further decorated by oxidation and acylation reactions. Similar to tubulysin, pretubulysin exhibits also highly valuable activities inducing apoptosis and inhibiting cancer cell migration and tubulin assembly in vitro and in vivo (Herrmann et al. 2012; Braig et al. 2014). The *tub* cluster was inserted into the chromosome of *P. putida* using the MycoMar transposon and was expressed relying on the native promoter(s) and by additional insertion of the P_*tet*_ promoter from transposon Tn10 in front of *tubCDEF*. This resulted in the production of the two variants *pretubulysin A* (Fig. 1) and *tyrosine pretubulysin A* (a product of module skipping). Yields could be pushed eightfold to 1.76 μg/l by supplementing pipecolic acid. Interestingly, in contrast to observations made with myxochromide S, yields of pretubulysin A were doubled at 30 °C compared to 16 °C cultivation temperature.

Two members of the syrbactin group that are synthesized by type I PKS/NRPS hybrid systems (Dudler 2014) were produced in *P. putida* P3. The compounds have received particular attention for their ability to inhibit proteases and induce apoptosis in different cancer cell lines such as neuroblastoma (Archer et al. 2010). *Syringolin A* (Fig. 2, **7**) was produced by expression of the *sylABCDE* cluster from *Pseudomonas syringae* pv. *syringae* (Ramel et al. 2009). The biosynthesis core is constituted by *sylC* encoding an NRPS module and *sylD* encoding two NRPS modules and one PKS module. Syringolin A is synthesized from valine which is joined to a second molecule valine by an unusual ureido group, 3,4-dehydrolysine, another valine and malonyl-CoA. SylB is thought to act as desaturase converting lysine to 3,4-dehydrolysine, while *sylA* and *sylE* encode a putative transcription activator and the exporter of syringolin. The 22-kb gene region was expressed from a cosmid relying on the original promoters. Produced syringolin A was recovered from the medium, corroborating functionality of the syringolin exporter encoded in the cluster. *Glidobactin A* was produced by expression of the homologous *plu1881*–*1877* gene cluster from *Photorhabdus luminescens* subsp. *laumondii* TT01 (Dudnik et al. 2013). The biosynthesis core in the 18.3-kb cluster consists of NRPS encoding *plu1878* (*sylC* homolog) and NRPS/PKS encoding *plu1880* (*sylD* homolog). In glidobactins, the ureido-valyl moiety typical for syringolin is replaced by a fatty acid tail attached to the starter amino acid threonine. Glidobactin A biosynthesis proceeds by incorporation of lysine, which is modified by 4′-hydroxylation, alanine, and malonyl-CoA. Remarkably, heterologous production in *P. putida* was successful in unagitated cultures, grown for 5 days. Determination of product yields was not in focus of the study.

*Prodigiosin* (Fig. 2, **8**) represents a metabolite derived from a biosynthesis pathway very different from the typical NRPS/PKS assembly lines but still related via some shared motives in the involved enzymes. The red colored tripyrrolic pigment with highly valuable bioactivities such as antibiosis and cytotoxicity (Hassankhani et al. 2014; Lapenda et al. 2014) is synthesized from 2-octenal, pyruvate, proline, malonyl-CoA, and serine in a complex bifurcated pathway, whose two products are condensed in a final reaction to form prodigiosin (Williamson et al. 2006). In *S. marcescens* W838, the pathway is encoded in the 22-kb *pigABCDEFGHIJKLMN* cluster. PigA, PigG, PigH, PigI, and PigJ act together as an NRPS/PKS assembly line producing a chain from proline, malonyl-CoA, and serine (Garneau-Tsodikova et al. 2006). Application of the TREX expression system enabled identification of *P. putida* KT2440 as a promising producer by chromosomal integration and P_*T7*_*-*dependent expression of the *pig* gene cluster, yielding 0.5 mg/g cell dry weight (CDW) of prodigiosin (Loeschcke et al. 2013). Based on that, the group evaluated a different expression setup, pushing yields by 2 orders of magnitude (Domröse et al., unpublished) and proving the capability of *P. putida* for high-level production of the interesting metabolite. Here, the expression strategy appeared to be crucial for increasing yields.

The general capability of *P. putida* to synthesize PKS/NRPS products is undisputed as documented by the here provided list of successful examples. As mentioned above, the bacterium’s features such as its PPTase and xenobiotic tolerance render it especially suitable for the production of this group of compounds. Thus, use of *P. putida* for expression of PKS/NRPS systems can be considered as a valuable option for the identification of high-value compounds and the elucidation of their biosynthetic pathways. However, yields are often low and compounds are too diverse to recommend a general strategy for improvement. Considering the large gene clusters encoding biosynthesis, the expression strategy is surely a dominant bottleneck. On the level of biosynthesis, it is worth underlining that in some cases, cultivation conditions significantly influenced production, apparently in a highly product-specific manner. Optimal conditions thus have to be evaluated experimentally. Since several studies report increased yields if precursors are supplemented, engineering approaches to enhance precursor supply may in the future improve production.

## Further products from amino acid metabolism

### Aromatic compounds

The extraordinary tolerance *of P. putida* S12 towards organic solvents (Weber et al. 1993; Kuepper et al. 2015) enabled its application in the production of various aromatic compounds valuable as building blocks for bioactive small molecules, resins, and polymers (Meijnen et al. 2011a). Here, shikimate pathway-derived amino acids are utilized as precursors (Gosset 2009).

In contrast to many of the compounds described in this review, introduction of only one or two enzymes instead of whole operons or gene clusters into *P. putida* is sufficient for the production of aromatics. Accumulation of diverse products was implemented by additionally targeted control of the host’s intrinsic metabolic inventory by deletion of genes at different stages of aromatics catabolism. This way, several aromatics producers were generated and developed from one another. An excellent overview on the genealogy of the strains applied for production of aromatics is given by Tiso et al. (2014). The relevant studies are summarized briefly in the following.

First, *t-cinnamate* and *phenol* producer strains were generated by introduction of the phenylalanine/tyrosine ammonia lyase gene *pal* from *Rhodosporidium toruloides* ATCC 64815 and the tyrosine phenol lyase gene *tpl* from *Pantoea agglomerans* AJ2985, respectively (Nijkamp et al 2005; Wierckx et al 2005). Heterologous expression of these key biosynthetic enzymes was controlled by the salicylate-inducible promoter system NagR/P*nag*Aa from *Comomonas testosteroni* (*tpl*) or P_*tac*_ (*pal*) and resulted after further strain improvement by chemical mutagenesis in production strains that accumulated extracellularly 5.4 mM (0.8 g/l) *t*-cinnamate and 9.2 mM (0.9 g/l) phenol, respectively.

Based on the abovementioned two chassis strains, producers of further aromatic compounds were constructed. Nijkamp et al. (2007) enhanced metabolic flux towards tyrosine and introduced phenylalanine auxotrophy by random mutagenesis of the *t*-cinnamate producer strain. Additional deletion of the feruloyl-CoA synthetase gene *fcs* whose product catalyzes the first step of *p-*coumarate degradation led to an effective production strain accumulating *p-coumarate* (Fig. 1) instead of *t-*cinnamate in amounts of up to 1.7 g/l.

A *p-hydroxystyrene* producer was constructed by additional introduction of the *p-*coumaric acid decarboxylase gene *pdc* from *Lactobacillus plantarum* DSM20174 into the *fcs*-deficient *pal* expressing chassis strain with yields up to 21 mM (2.5 g/l) with expression of *pal* and *pdc* under NagR/P*nag*Aa control (Verhoef et al. 2009). Accumulation of *p-hydroxybenzoate* up to 1.8 g/l was achieved by Verhoef et al. (2007) via replacement of the *tpl* expression vector in the phenol producer strain by a *pal* expression vector and deletion of *pobA* in the host’s genome. The latter gene product catalyzes the first step in *p*-hydroxybenzoate degradation. As a result, Pal-synthesized *p*-coumarate was converted by *P. putida* S12 intrinsic metabolic pathways to *p*-hydroxybenzoate, which was not further degraded. Comparison of transcriptome data from the producer strain with the wild-type *P. putida* S12 identified the multidrug efflux MFS transporter PP1271–PP1273 to play an important, but not essential, role in *p*-hydroxybenzoate secretion. Furthermore, the 4-hydroxyphenylpyruvate dioxygenase encoding gene *hpd* as a target for further strain improvement. Deletion of *hpd* enhanced metabolic flux towards the product and led to an increase in yield from 0.25 to 0.31 g/l (2.3 mM) in the applied experimental setup by Verhoef et al. (2010). Targeted engineering of precursor supply for aromatic molecules via the pentose phosphate pathway was achieved by introduction of the *E. coli* DH5α xylose degradation genes *xylAB*_*FGH*. The respective enzymes channeled xylose into the pathway which resulted in yields increased by further 22 % and moreover enabled production of these compounds from plant biomass (Meijnen et al. 2011a, 2011b).

The listed examples and convincing yields show the potential for *P. putida*-based industrial production processes. Besides engineered enhancement of the precursor pool, random chemical mutagenesis, and subsequent application of feasible screening strategies proved to be excellent tools for simple generation and identification of strains with improved aromatics production (Tiso et al. 2014). In some of the mentioned studies, optimal yields of aromatics were obtained by application of two-phase systems which combine the culture medium with an organic solvent for immediate removal of toxic aromatic products, a technique for which *P. putida* S12 is especially suited due to its remarkable solvent tolerance (Heipieper et al. 2007, Verhoef et al. 2009).

Aromatic amino acid-derived metabolites other than phenolic compounds that were produced in *P. putida* are *N-acyl aromatic amino acids*, a class of secondary metabolites with antimicrobial activity that was initially discovered by screening of metagenomic libraries for compounds capable of inhibiting *Bacillus subtilis*. Active expression of the biosynthetic machinery with the key enzymes from the *N*-acyl amino acid synthase family from a 29-kb environmental DNA fragment and secretion of the product was reported for *P. putida* KT2440 (Craig et al. 2010)*.* In the same study, introduction of environmental DNA was sufficient to induce increased intrinsic production of porphyrine-derived pigments in clones of a metagenomic library in *P. putida*. Therefore, the authors conclude that *P. putida* has a high potential in metagenome-based studies concerning the detection of novel secondary metabolites. Considering that *P. putida* does not intrinsically produce antibiotics but exhibits extraordinary tolerance to producing such compounds, it may prove especially useful in functional screenings for novel antimicrobials. This potential is yet to be exploited. Further reports support the idea of *P. putida* as an alternative host for functional screening of metagenomics libraries (Troeschel et al. 2012; Liebl et al. 2014).

Likewise an aromatic amino acid derivative is the bacterial pigment *deoxyviolacein* (Fig. 2, **9**). It is formed from two molecules of tryptophan and described to show dyeing and antimicrobial properties (Xing and Jiang 2011; Jiang et al. 2012). This derivative of violacein was accumulated in *P. putida* mt-2 at levels of 1.5 g/l dependent on expression of *vioABCE*, an engineered variant of the violacein biosynthesis operon *vioABCDE* from *Duganella* sp. B2, controlled by the alkane-inducible *alKB* promoter from *P. putida* (Xing and Jiang 2011).

In bacteria, the shikimate pathway provides precursors like chorismate not only for mentioned aromatic amino acids but also for different carbocyclic aromatic secondary metabolites like phenazines. These often antimicrobial redox mediators fulfill different functions, e.g., to support virulence and competitive fitness of the producing organisms. Due to their antibacterial and antifungal properties, phenazine derivatives are of interest for pharmaceutics and biocontrol (Pierson and Pierson 2010; Jayaseelan et al. 2014). Glandorf et al. (2001) modified the plant growth promoting *P. putida* strain WCS358r to produce the yellow pigment PCA (*phenazine1*-*carboxylic acid*) by P_*tac*_-controlled expression of the genome-integrated *phzABCDEFG* operon from *P*. *fluorescens* 2-79. Thereby, the host strain was turned into an improved fungal growth-inhibiting biocontrol strain (Glandorf et al. 2001; Bakker et al. 2002; Viebahn et al. 2005). Recently, production of PCA and its subsequent conversion to *pyocyanin* (Fig. 2, **10**) was established in KT2440 by simultaneous expression of *phzA1B1C1D1E1F1G1* and *phzMS* from *P. aeruginosa* PAO1 on two compatible plasmids (Schmitz et al. 2015), yielding 45 mg/l pyocyanin. Remarkably, accumulating the recombinant redox mediator in the culture medium enabled *P. putida* to sustain strongly oxygen-limited culture conditions by redox balancing via an anode as electron acceptor.

These examples suggest *P. putida* to be a suitable host for production of aromatic pigments with antimicrobial activity in future studies.

### Non-aromatic compounds

Besides these examples for utilization of aromatic amino acids or their precursor molecules, it was demonstrated that other amino acids provided by the bacterium’s metabolism can also serve as precursors for different natural products.

*Monoethanolamine***(**MEA) is an alkanolamine extensively used, e.g., to prevent corrosion, as a detergent, as a precursor for the production of ethylamines, or for CO_2_ capture (Aaron and Tsouris 2005; Foti et al. 2013). Currently, most MEA is produced from petroleum feedstock. A sustainable alternative was offered by production of MEA in the solvent tolerant *P. putida* S12 strain via decarboxylation of serine (Foti et al. 2013). To this end, the authors introduced l-serine decarboxylase (*sdc*) genes from *Arabidopsis thaliana* (ecotype Columbia) and *Vibrio carteri f. nagariensis*. Efficient formation of MEA was only observed with a truncated version of *A. thaliana* Sdc. Strain improvement by deletion of genes for MEA breakdown (*eutBC*) and media optimization led to yields up to 0.2 g/l (2.6 mM in culture medium).

*Cyanophycin* is a polymer consisting of arginine and aspartic acid, which was initially discovered in cyanobacteria (Frommeyer et al. 2014). Compounds derived thereof have gained interest for their applicability in technical processes and as pharmaceuticals (Mooibroek et al. 2007; Steinbüchel and Sallam 2010). Consequently, several studies aimed at recombinant production to achieve improved biotechnological access to this polyamide (Frommeyer et al. 2014). *P. putida* KT2440 was first used for constitutive expression of the cyanophycin synthetase *cphA* from *Synechocystis sp.* strain PCC6308. In mineral salt medium supplemented with aspartic acid and arginine, the product could be accumulated to 11 % of CDW. However, the obtained polymer exhibited reduced length and polydispersity compared to the authentic material from cyanobacteria (Aboulmagd et al. 2001). Voss et al. (2004) expressed *cphA* from further cyanobacterial strains in KT2240 as well as GPp104, which resulted in the accumulation of cyanophycins to amounts between 6.8 and 24 % of CDW in mineral media. Highest yields were achieved by expressing the cyanophycin synthetase of *Anabena* sp. strain PCC72120. GPp104 being deficient for the production of the intrinsic polymer PHA (see above) accumulated in general slightly higher amounts than the wild type during expression of any CphA. Interestingly, substitution of arginine residues by lysine within the polymer up to 10 mol% was observed in case of cphA_6308_, if arginine was not supplemented to the mineral medium. Another modified variant of cyanophycin with citrulline partially substituting arginine was produced by choosing the citrulline accumulating strain *P. putida* ATCC 4359 as expression host (Wiefel et al. 2011). Optimization of culture conditions resulted in total amounts of citrulline-containing cyanophycin of 43.4 % of CDW. Partial substitution of arginine by other amino acids like lysine or citrulline was shown to lead to increased solubility of the naturally largely insoluble polymer (Frommeyer and Steinbüchel 2013).

## Perspectives

*P. putida* has been biotechnologically domesticated by concerted efforts of different fields of modern microbiology, especially over the last decade. The here given overview of natural products that could be synthesized by heterologous gene expression demonstrates the bacterium’s potential in this context. Synthetic biology further spurs these developments: Recent studies reporting a genome-edited *P. putida* strain provide a robust next-generation cell factory with enhanced features regarding genetic stability as well as energy state and availability of reduction equivalents for future studies (Martínez-García et al. 2014b; Lieder et al. 2015). Furthermore, liberation of *P. putida* from its obligate aerobic nature has been reported, providing the basis for bioreactor-based processes without aeration and allowing natural product formation by oxygen-sensitive biocatalysts (Nikel and de Lorenzo 2013; Schmitz et al. 2015). Engineering the bacterium for efficient utilization of lignin-derived aromatics and xylose expanded the spectrum of applicable carbon sources and enables the utilization of plant biomass (Meijnen et al. 2011a; Meijnen et al. 2012; Johnson and Beckham 2015). In such advanced cell factories (Fig. 3), novel cloning and expression technologies will allow implementation of various biosynthetic production pipelines, further expanding *P. putida*’s product portfolio (Zhang et al. 1998; Fu et al. 2008, Gibson 2011; Martínez-García and de Lorenzo 2011; Fu et al. 2012 Martínez-García and de Lorenzo 2012; Loeschcke et al. 2013; Durante-Rodríguez et al. 2014; Martínez-García et al. 2014a). By in-depth analysis of the bacterium’s carbon metabolism as well as application of available “omics” tools, a systems biology perspective will provide the basis for knowledge-based metabolic engineering of *P. putida* in order to increase yields of natural products (Nogales et al. 2008; Puchałka et al. 2008; Wu et al. 2011; Sudarsan et al. 2014; Simon et al. 2014). This ever-accelerating development in methodology and knowledge gain paves the way for the research to come in the field of natural product biosynthesis with *P. putida*.

Future opportunities for application of *P. putida* may include the production of valuable compounds, but also the use as delivery system for a variety of bioactive molecules (Fig. 3). In this context, the bacterium’s outer membrane vesicles are discussed for their potential as adjuvants or vaccine carriers due to their low pathological activity (Choi et al. 2014). Furthermore, based on release of intrinsically or recombinantly produced compounds, it was suggested to apply *P. putida* for plant growth promotion and protection of plants from disease as biocontrol agent (Bakker et al. 2002; Glick 2012). Likewise, potential application of engineered *P. putida* in the context of soil remediation via delivery of suitable metabolites or enzymes was pointed out (de Lorenzo 2008; Cao et al. 2012; Chen et al. 2013).

Such studies demonstrate the potential of *P. putida* in highly diverse fields of application and may inspire further exciting developments towards the establishment of *P. putida* as a platform for production of various natural products in the future.
